# Breastmilk intake among exclusively breastfed Indonesian infants is negatively associated with maternal fat mass

**DOI:** 10.1038/s41430-019-0458-1

**Published:** 2019-06-24

**Authors:** Aly Diana, Jillian J. Haszard, Lisa A. Houghton, Rosalind S. Gibson

**Affiliations:** 1 Department of Human Nutrition, University of Otago, Dunedin, New Zealand; 2 Faculty of Medicine, Universitas Padjadjaran, Bandung, Indonesia

**Keywords:** Nutrition, Paediatrics

## Abstract

Excessive maternal fat mass may impair lactogenesis and lead to lower breastmilk volume. We investigated this relationship in rural Indonesian exclusively breastfeeding mother–infant (2–5.3 months) dyads (*n* = 112) by measuring breastmilk intake by deuterium oxide dose-to-mother technique (DDMT) and maternal fat mass by DDMT, bioelectrical impedance analysis (BIA), and body mass index (BMI). We also compared fat mass assessed by DDMT and BIA. In this population, we found a significant negative relationship between breastmilk intake and maternal fat mass measured by DDMT (*β* = −5.04 mL, 95% CI: −9.36, −0.72, *P* = 0.023), and similar but slightly weaker negative trend with BIA and BMI, after adjusting for social-economic status, maternal age, infant age and sex. Maternal fat mass estimates by BIA and DDMT showed good agreement. In light of the trend for overweight and obesity worldwide, further research is needed into the underlying mechanisms of this negative relationship.

## Introduction

Exclusive breastfeeding up to the first six months of postnatal life is a WHO recommendation. However, excessive body fat is said to be associated with a less adequate milk supply and significantly lower rates of initiation, duration, and exclusivity of breastfeeding, irrespective of the women’s ethnicity [1–3]. The cause of such poor lactation performance is said to be multi-factorial, and may include biological, psychosocial and mechanical factors [3, 4].

Most investigations of the relationship between poor lactation performance and excessive maternal body fat have used pre-pregnant body mass index (BMI) to classify women as overweight or obese, even though BMI fails to distinguish between weight associated with muscle with weight from body fat. Furthermore, BMI has often been calculated from self-reported pre-pregnancy weight and height [1], a practice that can lead to bias and further compromise the reliability of the data. Few studies have applied the deuterium oxide dose-to-mother technique (DDMT) to generate simultaneously precise information on both breastmilk intake in exclusively breastfed infants and the degree of fatness of their mothers [2]. Here, we have investigated this relationship in a group of rural Indonesian mother–infant dyads with breastmilk intake measured by DDMT, and maternal fat mass by DDMT, bioelectrical impedance analysis (BIA) or BMI. We have also compared fat mass (as percent) measured by DDMT and BIA.

## Methods

### Participants and methods

Rural Indonesian mother–infant (2–5.3 months) dyads (*n* = 121) from Sumedang district, West Java, Indonesia were purposively recruited for this cross-sectional study, as described earlier [5]. Of these, 9 mother–infant dyads were excluded because of implausible breastmilk intake (*n* = 1) or fat mass (*n* = 1) and failure to comply to exclusive breastfeeding based on 6-days of in-home observations (*n* = 7). Ethical approval was obtained from the Human Ethics Committees of *Universitas Padjadjaran*, Indonesia, and the University of Otago, New Zealand. Informed consent was obtained from all subjects.

Socio-demographic data and maternal weight and height were collected by trained research assistants. Breastmilk intake of exclusively breastfed infants and maternal fat mass were each assessed via DDMT over 14 days, following the International Atomic Energy Agency (IAEA) protocols, including conditions controlling for pre-measurement behaviours that may alter hydration state [6]. In brief, the deuterium enrichment in each saliva sample was measured by fourier transform infra-red spectrometry (FTIR); and the results were entered into an IAEA spreadsheet (Microsoft Excel 2013) to calculate milk intake and maternal fat mass by fitting the curve using the imbedded equations. Maternal fat mass was also assessed using BIA (Tanita SC-240MA, Itabashi-ku, Tokyo, Japan). Maternal BMI was calculated from post-partum measurements of weight and height using the WHO classification for overweight (≥25 BMI <30) and obesity (BMI ≥30) for non-pregnant, non-lactating women [7].

### Statistical analyses

All continuous variables were assessed for normality. Participant characteristics are expressed as mean ± s.d. Multivariate regression analyses assessed the association between breastmilk intakes and maternal fat mass measured by either DDMT, BIA, or BMI (adjusted for socio-economic status, maternal age, infant age, and infant sex). Agreement between maternal fat mass assessed by DDMT and BIA was assessed by Bland–Altman plots [8]. Analyses were performed using Stata version 12.0 (StataCorp LP, Texas, USA). *P* values < 0.05 were considered significant.

## Results

A total of 112 mother–infant dyads completed the study. Mean ages were 25.8 ± 6.1 years for mothers and 3.3 ± 0.8 months for infants, of whom 51.8% were female. Mean breastmilk intake was 787 ± 149 mL/day. Maternal BMI was 24.0 ± 3.8 kg/m^2^, with 29 mothers classified as overweight (BMI 25–29.9 kg/m^2^), 9 as obese (BMI≥30 kg/m^2^), 6 as underweight (BMI < 18.5 kg/m^2^), and the remainder as a healthy weight (18.5–24.9 kg/m^2^).

**Fig. 1:**
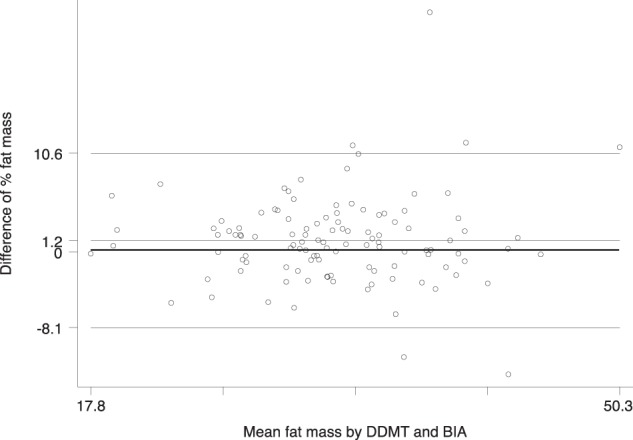
Bland Altman plot of maternal fat mass measured by deuterium oxide dose-to-mother technique (DDMT) and bioelectrical impedance analysis (BIA)

A significant negative relationship existed between breastmilk intake and fat mass as measured by DDMT (*β* = −4.88 mL, 95% CI: −9.59, −0.18, *P* = 0.042), with similar but slightly weaker relationships with fat mass measured by BIA and BMI (Table 1). The mean differences between DDMT and BIA for fat mass were small (1.2%). The Bland–Altman plot (Fig. 1) shows that agreement between the two measures was consistent across varying fat mass and that 95% limits of agreement were reasonably close (95% CI: 0.3–2.1%).

**Table 1:** Maternal fat mass and the association with breastmilk intakes

	Mean difference in breastmilk intake per unit increase (95% CI)				
	Mean ± s.d. (*n* = 112)	Unadjusted (*n* = 112)	*P*-value	Adjusted^a^ (*n* = 112)	*P*-value
BMI (kg/m^2^)	24.0 ± 3.8	−6.1 (−12.3, 0.1)	0.054	−4.4 (−12.1, 3.2)	0.255
Fat mass (BIA) (%)	33.2 ± 6.2	−4.4 (−8.5, −0.3)	0.035	−3.5 (−8.8, 1.9)	0.200
Fat mass (DDMT) (%)	33.4 + 6.3	−5.6 (−9.6, −1.5)	0.008	−4.9 (−9.6, −0.2)	0.042

^a^adjusted for socio-economic status, maternal age (years), infant age (months), and infant sex

*CI* confidence interval, *BMI* body mass index, *BIA* bioelectrical impedance analysis,

*DDMT* deuterium oxide dose-to-mother technique

## Discussion

Our results confirm a negative association between breastmilk intakes and maternal fat mass, irrespective of the method used to assess fat mass, with and without adjustment for socio-economic status, maternal age (years), infant age (months) and infant sex. Nonetheless, this negative relationship was strongest when DDMT was used for both measurements. Moreover, our BIA equipment provided a reliable estimate of maternal fat mass at 2.0–5.3 months post-partum in these exclusively breastfeeding mothers when compared with DDMT, highlighting the potential of this specific BIA model as a field tool. However, whether alternative BIA instruments employing different prediction equations have the potential to generate data on maternal fat mass of lactating women that is also reliable is uncertain.

Unfortunately, human studies designed to elucidate the underlying mechanisms of an inverse association between maternal fat mass and breastmilk volume are limited with equivocal results [3]. Here, our Indonesian mothers 2–5.3 months postpartum were all currently exclusively breastfeeding. As a consequence, biological factors such as differences in breast milk fat concentrations, hormonal imbalances and/or mammary hypoplasia are probably more likely to be responsible for the negative relationship observed here between maternal fat mass and breastmilk intake. Mechanical and psychosocial factors are said to be more likely to impact on early lactation performance [3].

Our study was based on a larger sample of exclusively breastfed infants (*n* = 112) than all earlier studies to date [1], and quantified breastmilk volume using DDMT, an approach which does not interfere with usual feeding practices, unlike test-weighing or maternal breastmilk expression. Moreover, our mean BM intake measured by DDMT for these exclusively breastfed infants is comparable to values reported globally [5]. The DDMT had the added advantage of generating precise information on maternal fat mass. However, we applied the WHO BMI classification for non-pregnant, non-lactating adults [7], even though our BMI measurements were on lactating mothers because of the absence of body composition reference data for lactating women [9]. Finally, our findings fail to extend our understanding of the underlying mechanisms influencing the relationship between breastmilk intake and maternal fat mass in this population because of the absence of accurate measurements of breast milk fat concentrations, as well as data on hormonal responses and mammary hypoplasia impacting later breastfeeding.

In conclusion, we have confirmed the existence of a negative relationship between maternal fat mass and intake of breast milk in exclusively breast fed infants less than 6 months of age. More research is needed to establish the underlying mechanisms impacting later breastfeeding especially in view of the increasing prevalence of overweight and obesity among women worldwide.
